# HER2 exon 20 insertion mutations and myelosuppression in lung adenocarcinoma patient: a case report and response to trastuzumab deruxtecan

**DOI:** 10.1186/s13019-023-02181-w

**Published:** 2023-04-03

**Authors:** Bin Wang, Yang Song, Xin Yang, Chuan Chen

**Affiliations:** 1 Department of Cell Biology and Genetics, Chongqing Medical University, Chongqing, 400016 China; 2 Department of Oncology, Daping Hospital, Army Medical University, #10 Daping Changjiang Branch Road, Yuzhong District, Chongqing, 400042 China; 3 Department of Pathology, Daping Hospital, Army Medical University, Chongqing, 400042 China

**Keywords:** Lung adenocarcinoma, HER2 exon 20 insertion mutation, T-DM1, DS-8201, Myelosuppression

## Abstract

**Background:**

Human epidermal growth factor receptor 2 (HER2) mutations occur in 2% of lung cancers.

**Case presentation:**

In this report, we presented a case of an Asian female who was diagnosed with lung adenocarcinoma. NGS results indicated HER2 exon 20 insertion mutation and PET/CT results showed multiple metastases in lower lobes of both lungs. Thereafter, she was treated with chemotherapy alone, combination of chemotherapy and targeted therapy and immunotherapy. Due to progressive disease, she was then received DS-8201. Imaging data indicated partial response to DS-8201 and tumor marker values decreased significantly, suggesting good efficacy. Nevertheless, DS-8201 was discontinued because of the development of myelosuppression (grade 3). Finally, she died at home due to platelet deficiency, white blood cell (grade 4), granulocytopenia, intracranial hemorrhage and gastrointestinal hemorrhage.

**Conclusions:**

This was an important case since it exerted effective response to DS-8201. Meanwhile, myelosuppression is also found in the patient, which requires attention to pulmonary symptoms and careful monitoring.

## Introduction

Lung adenocarcinoma is the most common histological subtype of non-small cell lung cancer, comprising around 40% of all lung cancer cases [1]. Lung adenocarcinoma patients are usually diagnosed at an advanced stage with disseminated metastatic tumors [2], indicating the importance of successful development of novel approaches in cancer treatment.

Human epidermal growth factor receptor 2 (HER2) is a member of receptor tyrosine kinase of ERBB family, whose mutation has been reported in about 2%-3% of patients with lung adenocarcinomas [3]. The most common HER2 mutations are in-frame insertions in exon 20, including A775_G776insYVMA and G778_P780dup [4]. Although the potential role of HER2 as a therapeutic target has been investigated in cancer patients, clinical studies of trastuzumab in combination with chemotherapy, and even the selection of HER2 protein overexpression for targeted therapy, have yielded disappointing results [5].

Here, we highlighted a clinical female case of lung adenocarcinomas harboring HER2 exon 20 insertion mutations who experienced partial response to trastuzumab deruxtecan (DS-8201) after failure of chemotherapy, targeted therapy and immunotherapy.

## Case presentation

**Fig. 1:**
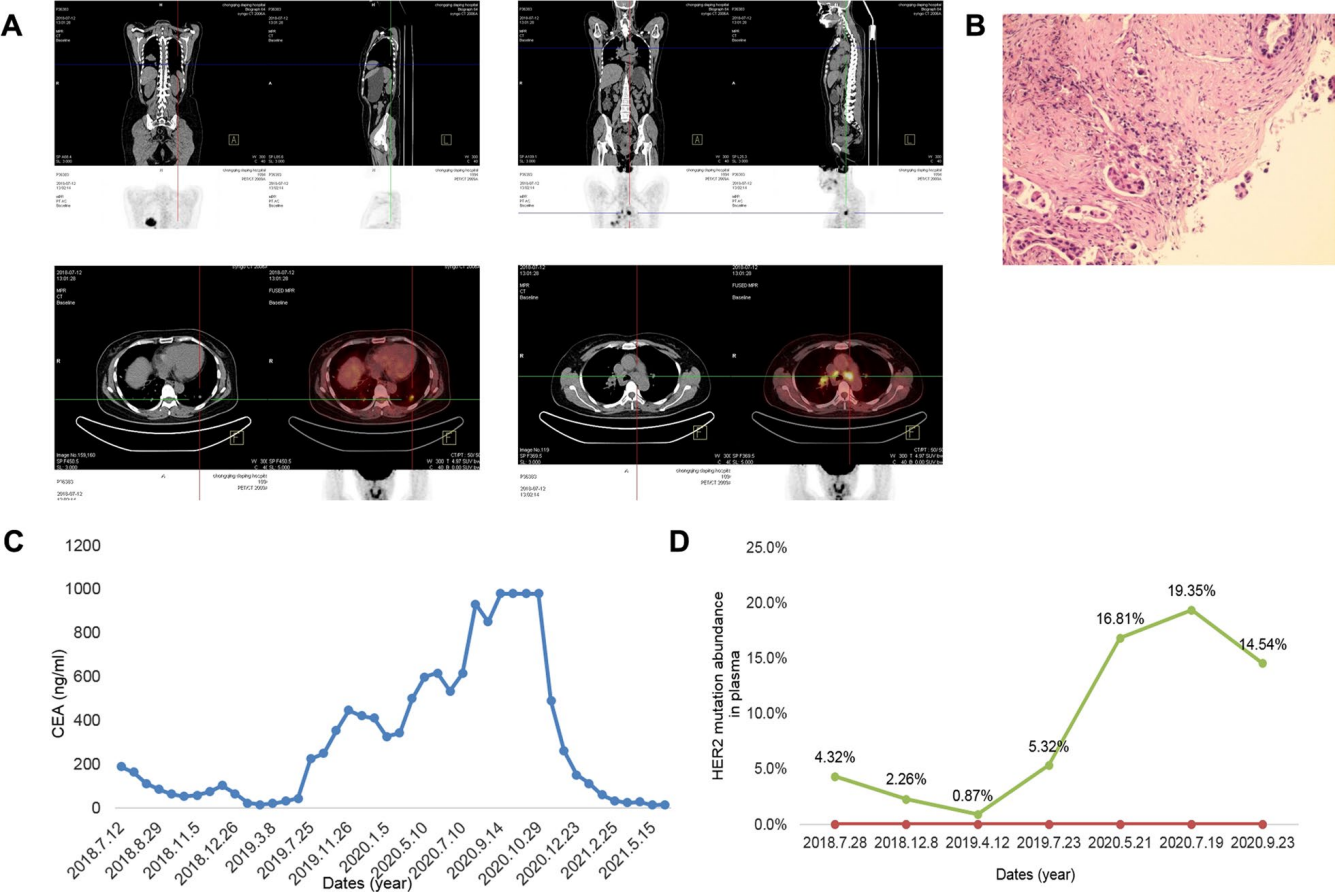
**A** Positron emission tomography/computed tomography (PET/CT) view (July 12th, 2018) of the whole body. **B** CT-guided core needle biopsy (July 16th, 2018) suggested lung adenocarcinoma. **C** HER2 exon 20 insertion mutation (p.Y772-A775dupYNMA) abundance in plasma. **D** The curve of carcinoma embryonic antigen after treatment

**Fig. 2:**
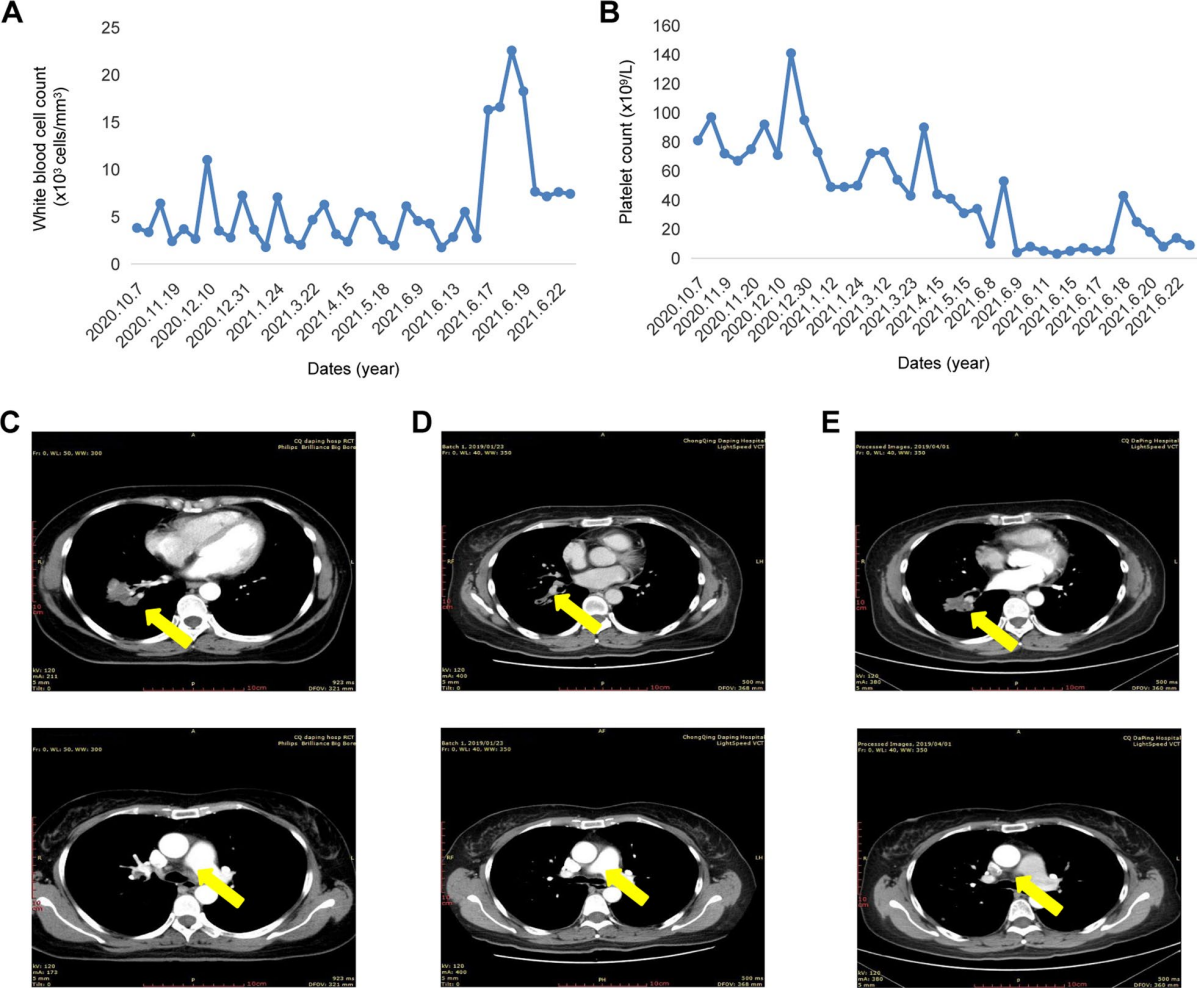
The curves of **A** white blood cell count and **B** platelet count after treatment. **C** Chest CT results (October 8th, 2018) showed that the mass image of the right lower lung was reduced, indicating partial response to Bevacizumab + Pemetrexed disodium + Carboplatin. **D**, **E** Chest CT results (December 27th, 2018 and April 1st, 2019) showed enlarged bilateral lung lesions, with no significant changes in lymph nodes

A 50-year-old Chinese woman with a history of chronic viral hepatitis B for more than 20 years suffered from asthma and cough with hemoptysis in July, 2018. No symptoms such as chest pain, chest tightness, fever, loss of weight or shivering were observed. On July 12^th^, 2018, she presented to our hospital. Positron emission tomography/computed tomography (PET/CT) results showed as follows (Fig. 1A): (a) mass soft tissue density shadow in the lower lobe of the right lung, abnormal increase of fluoro-D-glucose (FDG) metabolism, indicating peripheral lung cancer; (b) multiple metastases in lower lobes of both lungs; and (c) multiple lymph node enlargement and metastasis in cervical root of both lungs, hilum of right lung and mediastinum. Cranial enhanced nuclear magnetic resonance imaging (MRI) showed scattered ischemic foci in bilateral subfrontal cortex. CT-guided percutaneous core needle biopsy of the right lung suggested lung adenocarcinoma (Fig. 1B), accompanied by increase of tumor markers, including cytokeratin 19 fragment (CYFRA21-1, 17.03 ng/ml), carbohydrate antigen 12–5 (CA12-5, 143.14 U/ml), carcinoma embryonic antigen (CEA, 188.42 ng/ml) (Figure) and CA 19–9 (931.46 U/ml) (Fig. 1C). Then, next-generation sequencing (NGS), which covers exon regions of 550 cancer-related genes, was performed on DNA derived from the tumor biopsy specimen. Meanwhile, Pemetrexed disodium (0.4 g VD on day 1) for one cycle was initiated on July 21th, 2018. Then she was treated with Bevacizumab + Pemetrexed disodium + Carboplatin for 5 cycles based on NGS results [HER2 exon 20 insertion mutations (p.Y772-A775dupYNMA) and TP53 p.G226D], with mutation abundance of 4.32% (Fig. 1D). After treatment, decreased white blood cells and platelet counts (Fig. 2A, B) and partial response to the combined chemotherapy and targeted therapy were noticed (Fig. 2C).

However, the continuous combination therapy resulted in enlargement of bilateral lung lesions and values of tumor markers. Further elevated tumor marker values were found after the treatment with Bevacizumab + Albumin-bound Paclitaxel. Therefore, Trastuzumab (400 mg) and Nedaplatin (60 mg) were added. Re-examination results (December 12^th^, 2018) indicated that values of tumor markers reduced, suggesting that the combined therapy was effective. Whereas, the treatment was delayed for 1 week due to the development of myelosuppression (grade 3). Subsequently, Albumin-bound Paclitaxel was discontinued, followed by 4 cycles of Bevacizumab + Trastuzumab + Nedaplatin q3w, one cycle of Bevacizumab + Trastuzumab, and one cycle of Bevacizumab + Trastuzumab + Pertuzumab for continuation maintenance therapy. The re-examination of chest CT indicated that bilateral lung lesions were significantly smaller than before (Fig. 2D). MRI of the whole spine showed degeneration of the whole spine, and endplate inflammation was considered (stage II).

**Fig. 3:**
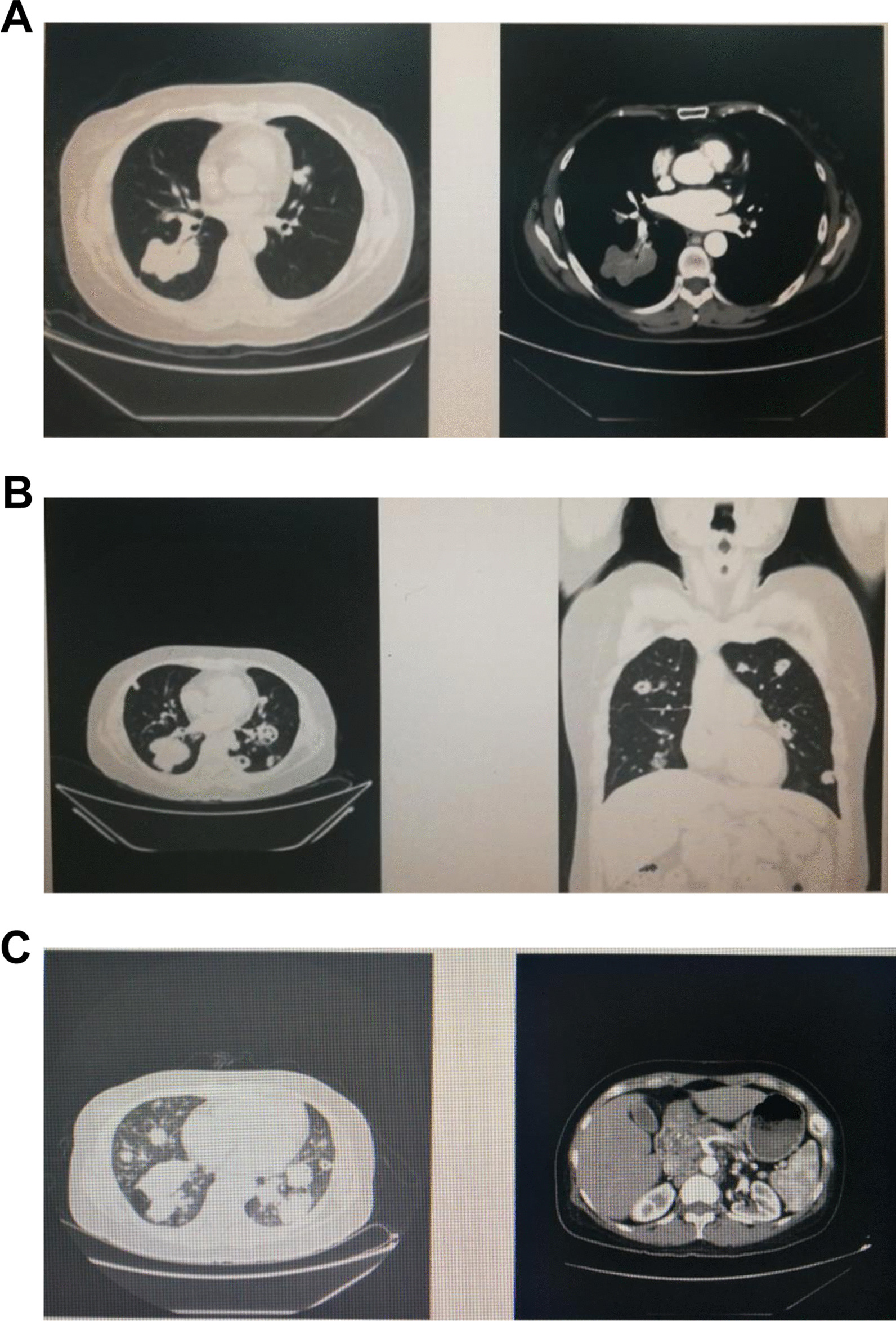
**A** CT results (December 20th, 2019) indicated that some bilateral lung nodules were slightly reduced, and some nodules showed small cavities, suggesting progressive disease. **B** CT results (May 10th, 2019) suggested increased and enlarged bilateral lung nodules and some cavities appeared. **C** CT results (September 14th, 2020) showed increased and enlarged bilateral lung nodules, as well as enhanced cervical and thoracic lesions

Since February 16th, 2019, the patient was treated with Bevacizumab + Trastuzumab + Nedaplatin. Due to the slow progress of the disease treatment, with HER2 mutation abundance of 0.87% (Fig. 1D, 2E), she was treated with Poziotinib 16 mg for 4 cycles. During treatment, the patient progressed from an initial stabilization of the right lower lung and reduced left clavicular lymph nodes to progressed left lung lesion, with no lesion in the skull. She also had severe paronychia and oral ulcers. On December 12th, 2019, the patient received Pyrotinib + Nivolumab for one cycle, followed by the addition of Anlotinib (12 mg) for another 2 cycles. Re-examination results of December 19th, 2019 showed larger double lung lesions and formation of bone metastases, suggesting progressive disease (Fig. 3A). She was treated with Pyrotinib + Anlotinib for 1 cycle, and Thymus facin (Ridaxian) for injection was used for anti-bone metastasis. On May 10, 2020, the double lung nodules were increased and enlarged, and cavities appeared in some nodules, indicating progressive disease (Fig. 3B); and peripheral blood NGS showed HER2 mutation p.Y772-A775dupYNMA with a frequency of 16.81%, accompanied by MDM2 copy number increase and TMB-L. Thus, the therapy changed to Trastuzumab + Pertuzumab + Bevacizumab + Nedaplatin for 2 cycles. In July 2020, the patient successively received targeted therapy with trastuzumab emtansine (T-DM1) 200 mg alone, TDM-1 + immunotherapy with NK cells (100 ml) once a week, TDM-1 combined with Pyrotinib for one cycle, and TDM-1 + Olaparib + Bevacizumab. CT showed increased and enlarged bilateral lung nodules, and increased lesions in cervical and thoracic vertebrae (Fig. 3C).

Since the values of tumor markers still elevated, DS-8201 (300 mg 3w/q) combined with NK cell therapy were initiated on October 7th, 2020. Due to myelosuppression (grade 3), the treatment regimen was adjusted to DS-8201 250 mg once every three weeks. Imaging data indicated partial response to DS-8201and tumor marker values decreased significantly, suggesting good efficacy. Nevertheless, DS-8201 was discontinued because of decreased platelet and white blood cell counts. Meanwhile, she was admitted to the hospital for transfusion of platelet therapy, altribopa, recombinant human thrombogenietin injection, Avattribopa maleate tablet, pegylated recombinant human granulocyte stimulating factor needle and other treatments. The patient resumed the treatment of DS-8201 from October 7th, 2020 to April 16th, 2021, and the imaging results showed that partial response to DS-8201 and tumor marker values decreased significantly. Finally, she stopped taking DS-8201 due to the reduction of platelet and white blood cell counts. On August 27th, 2021, she died at home due to platelet deficiency, white blood cell (grade 4) and granulocytopenia, intracranial hemorrhage and gastrointestinal hemorrhage.

## Discussion

Although many technologies have been developed, NGS still plays an important part in the accurate diagnosis and therapy of lung adenocarcinoma patients. In our case, HER2 exon 20 insertion mutations (p.Y772-A775dupYNMA) was detected in the plasma by using NGS. Previous study has revealed that patients harboring HER2 mutations often have various sites of metastases, the most common of which are lung, followed by adrenal metastases, bone and brain [5]. Likewise, the patient in this study experienced multiple metastases in lower lobes of both lungs.

Chemotherapy is the standard treatment for HER2-mutant subjects with advanced lung cancer and particularly pemetrexed-containing regimens is the most effective therapy for lung adenocarcinomas [6]. In the current study, our patient was first given Pemetrexed disodium, followed by the addition of Bevacizumab and Carboplatin based on the NGS results. HER2-targeted therapy is known to improve clinical outcomes in HER2-positive breast, colorectal and gastric cancers. Also, responses to poziotinib, Bevacizumab, Pertuzumab, Anlotinib and pyrotinib in cancer patients with activating HER2 mutations have been reported in many clinical trials [7–9]. Moreover, case series of chemotherapy combined with trastuzumab achieved a 50% response rate in lung cancer patients with HER2-activating mutations [10]. Unfortunately, the patient in this study received chemotherapy, targeted therapy and immunotherapy, and the effects were not satisfactory such as increased tumor markers or enlarged double lung lesions. This indicates that the optimal HER2 therapy for a specific HER2 molecular target has yet to be determined clinically.

Antibody–drug conjugates have emerged as a potent cancer treatment strategy that combines the ability of monoclonal antibody to specifically target tumor cells with the highly potent lethal capability of agents with payloads too toxic for systemic administration [7]. T-DM1, an HER2 antibody–drug conjugate, has been shown to significantly improve survival in HER2-mutang lung cancer [10]. In our case, due to slow progress of the disease treatment, the patient received TDM-1. Nevertheless, the frequency of HER2 mutation was increased by 16.81%, accompanied by enhanced and enlarged bilateral pulmonary nodules. Following treatment with DS-8201, the imaging data indicated partial response to DS-8201 and tumor marker values significantly reduced, revealing good efficacy. DS-8201 is a novel HER2-targeted antibody–drug conjugate incorporating a DNA topoisomerase I inhibitor [11], which has anti-cancer efficacy in clinical trials and anti-tumor immunity in mouse models [12]. The phase 2 clinical trail of Li et al. have supported the clinical benefit of trastuzumab deruxtecan in patients with HER2-mutant NSCLC, while 49% of patients had drug-related grade 3 or higher adverse events which must be carefully monitored [13]. Tamura et al. have clarified that DS-8201 exerts preliminary activity in T-DM1-pretreated HER2-positive breast cancer patients [14]. Meanwhile, they also noticed that all patients had at least one treatment-emergent adverse event, with anemia in 17%, neutropenia in 14%, leukopenia in 9%, and thrombocytopenia in 8%. Our patient stopped taking DS-8201 because of grade 3 myelosuppression (decreased platelet and white blood cell), which was consistent with previous data [15].

In this study, HER2 exon 20 insertion mutation (p.Y772-A775dupYNMA) is found in the lung adenocarcinomas patient and displays a partial response to DS-8201. Meanwhile, myelosuppression (grade 3) is also observed in the patient, which requires attention to pulmonary symptoms and careful monitoring.

## Data Availability

The data used to support the findings of this study are available from the corresponding authors upon reasonable request.
